# Dietary fat supplementation relieves cold temperature-induced energy stress through AMPK-mediated mitochondrial homeostasis in pigs

**DOI:** 10.1186/s40104-024-01014-7

**Published:** 2024-04-08

**Authors:** Wei He, Xinyu Liu, Ye Feng, Hongwei Ding, Haoyang Sun, Zhongyu Li, Baoming Shi

**Affiliations:** College of Animal Science and Technology, Northeast Agricultural University, 600 Changjiang Street, Harbin, 150030 PR China

**Keywords:** Apoptosis, Cold temperature, Energy stress, Fat, Glucolipid metabolism, Mitochondrial homeostasis

## Abstract

**Background:**

Cold stress has negative effects on the growth and health of mammals, and has become a factor restricting livestock development at high latitudes and on plateaus. The gut-liver axis is central to energy metabolism, and the mechanisms by which it regulates host energy metabolism at cold temperatures have rarely been illustrated. In this study, we evaluated the status of glycolipid metabolism and oxidative stress in pigs based on the gut-liver axis and propose that AMP-activated protein kinase (AMPK) is a key target for alleviating energy stress at cold temperatures by dietary fat supplementation.

**Results:**

Dietary fat supplementation alleviated the negative effects of cold temperatures on growth performance and digestive enzymes, while hormonal homeostasis was also restored. Moreover, cold temperature exposure increased glucose transport in the jejunum. In contrast, we observed abnormalities in lipid metabolism, which was characterized by the accumulation of bile acids in the ileum and plasma. In addition, the results of the ileal metabolomic analysis were consistent with the energy metabolism measurements in the jejunum, and dietary fat supplementation increased the activity of the mitochondrial respiratory chain and lipid metabolism. As the central nexus of energy metabolism, the state of glycolipid metabolism and oxidative stress in the liver are inconsistent with that in the small intestine. Specifically, we found that cold temperature exposure increased glucose transport in the liver, which fully validates the idea that hormones can act on the liver to regulate glucose output. Additionally, dietary fat supplementation inhibited glucose transport and glycolysis, but increased gluconeogenesis, bile acid cycling, and lipid metabolism. Sustained activation of AMPK, which an energy receptor and regulator, leads to oxidative stress and apoptosis in the liver; dietary fat supplementation alleviates energy stress by reducing AMPK phosphorylation.

**Conclusions:**

Cold stress reduced the growth performance and aggravated glycolipid metabolism disorders and oxidative stress damage in pigs. Dietary fat supplementation improved growth performance and alleviated cold temperature-induced energy stress through AMPK-mediated mitochondrial homeostasis. In this study, we highlight the importance of AMPK in dietary fat supplementation-mediated alleviation of host energy stress in response to environmental changes.

**Supplementary Information:**

The online version contains supplementary material available at 10.1186/s40104-024-01014-7.

## Introduction

Cold stress is one of the major challenges in animal husbandry, especially for family farms, during the autumn and winter at high latitudes and on plateaus [1, 2]. In mammals, nonshivering thermogenesis generates heat to maintain the body temperature and protect against cold temperature [3, 4]. In general, the increase in basal metabolism at low temperatures compels livestock animals to use more energy to maintain body temperature, which is the main reason for the decrease in productivity and increase in production costs [5, 6]. On the other hand, cold stress negatively impacts immunity, gut microbiota, and gut health in animals, thereby affecting growth and development [7, 8]. Therefore, a comprehensive understanding of the host’s metabolic state during cold temperatures and the identification of suitable nutritional strategies for improving productivity are necessary.

The energy diet is absorbed by intestinal epithelial cells after the emulsification of lipids and digestion by digestive enzymes, and transported to the liver after absorption via the small intestine to provide energy for the host [9–11]. As the central hub of energy metabolism, the liver determines the fate of substrates in response to the external environment and nutrient availability, which builds a high wall of energy homeostasis in the host [12]. Overall, the gut-liver axis is a key component in orchestrating host energy balance and metabolic homeostasis, and this axis responds rapidly to changing environmental conditions [13]. How cold temperature exposure reshapes energy metabolism in the host through the gut-liver axis has rarely been reported. AMPK, as an energy receptor and regulator, can regulate glycolipid metabolism to maintain energy homeostasis by activating phosphorylation during energy stress [14, 15]. As a cellular “power plant”, mitochondria produce ATP through the respiratory chain, but this process is accompanied by the generation of ROS [16]. Mitochondrial dynamics, including fusion and division, contribute to the mitochondrial respiratory chain and ROS scavenging [17]. However, interruption of mitochondrial dynamics can lead to mitochondrial fragmentation, swelling, disruption of the inner membrane, loss of cristae, and impaired respiration, which are classic hallmarks of apoptosis [18, 19]. Recently, AMPK was shown to mediate mitochondrial biogenesis and homeostasis to further induce endoplasmic reticulum (ER) stress, autophagy, and apoptosis [20–23]. Thus, for mammals experiencing energy stress at cold temperatures, AMPK may be a key target for coordinating host energy metabolism.

Based on the known role of energy stress, nutritional strategies for alleviating energy stress to improve productivity are urgently needed. As an essential component of mammalian tissues and diets, unsaturated fatty acids are involved in a variety of biological processes, such as the production of bioactive elements of cell membranes, the development of neurons and brain, and the production of precursors of cellular signalling molecules [24–26]. Omega-6 polyunsaturated fatty acids (n-6 PUFAs), which are a kind of PUFAs, are a major component of soybean oil and are considered a more feasible substitute for saturated fatty acids [27]. In recent years, several studies have shown that n-6 PUFAs could improve mitochondrial function, alleviate oxidative stress, increase membrane fluidity, and have beneficial effects on insulin sensitivity, blood lipids, and cardiovascular disease [27–30]. This evidence fully demonstrates the advantages of supplementation of soybean oil as an energy source at cold temperatures. However, the mechanism by which a high-fat diet alleviates energy stress in mammals at cold temperatures has rarely been illustrated. Here, we hypothesized that dietary fat supplementation can regulate glycolipid metabolism, mitochondrial function, and oxidative stress through AMPK and improve growth and feed efficiency in pigs. In the present study, we aimed to quantify the role and mechanism of dietary fat supplementation in improving growth performance and regulating energy metabolism and oxidative stress at cold temperatures, and to elucidate the relationship between dietary fat supplementation with mitochondrial biogenesis and homeostasis.

## Materials and methods

### Animals and experimental design

After a 3-d acclimatization period, 18 pigs (Duroc × Landrace × Yorkshire, 58 days old) were randomized into three experimental groups (*n* = 6/group, 3 gilts and 3 barrows) according to sex and initial BW (25.15 ± 0.43 kg). Specifically, all pigs were divided into two groups according to sex, stratified by BW, and further assigned to 1 of the 3 groups by stratified randomization. The 3 groups included: (1) the CON group: optimal temperature + basal diet (26.5 g/kg n-6 PUFAs), (2) the CL group: cold temperature + basal diet (26.5 g/kg n-6 PUFAs), and (3) the CH group: cold temperature + high-fat diet (40.9 g/kg n-6 PUFAs). Pigs in the CON group and CL/CH group were kept at 22 ± 3 °C and 14 ± 3 °C for 28 d, respectively. Moreover, the pigs were housed individually in stainless steel metabolic cages (1.5 m × 0.5 m × 0.8 m) and placed in the feeding room controlled by an electronic heater, and the temperature data were recorded by an electronic thermometer with a probe. Each metabolic cage was equipped with a feed trough and a drinking nipple. Pigs were fed three times a day (06:00, 11:00, and 16:00) and ensured that abnormal hunger did not occur until the next feeding. The animals were weighed on the 1^st^ and 28^th^ days of the experiment to evaluate their growth performance. In addition, the daily feed intake and health status of each individual were recorded daily. As shown in Table 1, the experimental diets were formulated according to the Chinese Standard GB/T 39235−2020 [31]. Moreover, the crude protein, amino acid, calcium, and available phosphorus to net energy levels of the diets were in fixed proportions. In this study, soybean oil and full-fat soybean meal were used as sources of n-6 PUFAs, and the content of n-6 PUFAs in the feed was determined as described previously [32].

**Table 1:** Ingredients and nutrient composition of the diets

Ingredients, %	Content, %	
	Basal diet	High-fat diet
Corn	72.30	62.50
Soybean meal	22.00	14.00
Full-fat soybean	0.00	15.00
Soybean oil	1.56	4.17
Dicalcium phosphate	1.20	1.34
Limestone	0.90	0.90
Salt	0.40	0.40
Lysine	0.43	0.45
Methionine	0.06	0.08
Threonine	0.12	0.13
Choline chloride	0.03	0.03
Premix^1^	1.00	1.00
Total	100.0	100.0
Nutrient levels^2^		
Metabolizable energy, Mcal/kg	3.20	3.42
Net energy, Mcal/kg	2.49	2.68
Crude fat	5.09	7.82
Crude protein	15.99	17.03
Lysine	0.98	1.06
Methionine	0.28	0.31
Threonine	0.60	0.63
Tryptophan	0.18	0.19
Calcium	0.66	0.71
Available phosphorus	0.26	0.28
Sodium	0.17	0.17
Chlorine	0.27	0.27

^1^Provided the following per kilogram of diet: Fe, 140 mg; Cu, 25 mg; Mn, 35 mg; Zn, 80 mg; Se, 0.4 mg; I, 0.5 mg; vitamin A, 10,000 IU; vitamin D_3_, 3,000 IU; vitamin E, 63 mg; vitamin K_3_, 3 mg; vitamin B_1_, 3 mg; vitamin B_2_, 9.6 mg; vitamin B_6_, 4.5 mg; vitamin B_12_, 0.04 mg; niacin, 36 mg; d-pantothenic acid, 30 mg; d-biotin, 0.24 mg; and folate, 1.8 mg

^2^Crude fat and crude protein were analyzed values and the rest were calculated values

### Sample collection

On d 28, the pigs were euthanized by electrical stunning after overnight fasting for 12 h. Blood was collected rapidly in heparin tubes, centrifuged (3,000 r/min, 10 min), and subsequently stored at –80 °C. The tissues of the liver and jejunum were washed with saline, and then, 1 cm samples were mixed with 4% formaldehyde and 0.5% glutaraldehyde fixatives and stored at 4 °C. The jejunal mucosa and ileal contents were collected quickly in sterile tubes and stored at –80 °C. At the same time, liver, jejunum, and ileum tissues were placed into sterile tubes and stored at –80 °C until analysis.

### Analysis of digestive enzyme activity

An appropriate amount of jejunal mucosal tissue was removed, saline (0.9% sodium chloride) was added at a ratio of 1:4, and the samples were homogenized using a tissue grinder (FSH-2A, Changzhou, China). The sample was subsequently centrifuged (2,500 r/min, 10 min) using a centrifuge (Universal 320R, Hettich, Germany), after which the supernatant was collected for the subsequent analysis. All the operations were performed at 4 °C. Alpha-amylase (C016-1-1), lipase (A054-1-1), trypsin (A080-2), sucrase (A082-2-1), maltase (A082-3-1), and lactase (A082-1-1) levels in jejunal mucosa were quantified using kits (Nanjing Jiancheng Bioengineering Institute, Nanjing, China) according to the manufacturer’s instructions.

### Analysis of plasma biochemistry

The plasma concentrations of total protein (TP, HY-N0011), globulin (GLO, HY-N0013), albumin (ALB, HY-N0012), glucose (GLU, HY-N0028), urea (HY-N0015), total bile acid (TBA, HY-N0044), low-density lipoprotein (LDL, HY-N0032), and high-density lipoprotein (HDL, HY-N0031) were analysed via commercial kits (Sino-UK, Beijing, China) using an automated biochemical analyser (Mindray BS-200, Shenzhen, China).

### Analysis of plasma hormones

Insulin (ML002341), glucagon (ML404356), and glucocorticoid (GC, ML414168) levels were quantified in plasma by ELISA kits (mlbio, Shanghai, China) using a microplate reader (Labsystems Multiskan MS, Helsinki, Finland).

### Untargeted metabolomic analysis

Untargeted metabolomic analysis of the ileal contents was conducted as described previously [33]. Briefly, a system that included ultra-high performance liquid chromatography (Waters, Acquity I-Class PLUS, Milford, MA, USA) with a high-resolution mass spectrometer (Waters, Xevo G2-XS Qtof, Milford, MA, USA) was used to analyze metabolites. For the collected data, Progenesis QI software was used to peak extraction and alignment, etc. The quality control samples and the repeatability of the samples within the group was determined using principal component analysis and spearman correlation analysis. The screening criteria of FC > 1, *P* < 0.05, and VIP > 1 were adopted to identify the differential metabolites.

### Cholesterol and triglycerides in the liver

An appropriate amount of liver tissue was removed, saline (0.9% sodium chloride) was added at a ratio of 1:4, and the samples were homogenized using a tissue grinder (FSH-2A, Changzhou, China). The sample was subsequently centrifuged (2,500 r/min, 10 min) using a centrifuge (Universal 320R, Hettich, Germany), after which the supernatant was collected for the subsequent analysis. All the operations were performed at 4 °C. Total cholesterol (T-CHO, A111-1-1) and triglycerides (TG, A110-1-1) levels in the liver were quantified using kits (Nanjing Jiancheng Bioengineering Institute, Nanjing, China) according to the manufacturer’s instructions.

### Analysis of glucose-metabolizing enzyme activity in the jejunum and liver

An appropriate amount of jejunum and liver tissue was removed, saline (0.9% sodium chloride) was added at a ratio of 1:4, and the samples were homogenized using a tissue grinder (FSH-2A, Changzhou, China). The sample was subsequently centrifuged (2,500 r/min, 10 min) using a centrifuge (Universal 320R, Hettich, Germany), after which the supernatant was collected for the subsequent analysis. All the operations were performed at 4 °C. Pyruvate kinase (PK, ML383325), pyruvate dehydrogenase (PDH, ML363240), citrate synthase (CS, ML333488), pyruvate carboxylase (PC, ML311258), and acetyl-coenzyme A (A-CoA, ML300128) were quantified in the jejunum and liver by ELISA kits (mlbio, Shanghai, China) using a microplate reader (Labsystems Multiskan MS, Helsinki, Finland).

### Analysis of oxidative stress in the jejunum and liver

A total of 5 markers of oxidative stress were selected to assess the oxidative status in the jejunum and liver. An appropriate amount of jejunum and liver tissue was removed, saline (0.9% sodium chloride) was added at a ratio of 1:4, and the samples were homogenized using a tissue grinder (FSH-2A, Changzhou, China). The sample was subsequently centrifuged (2,500 r/min, 10 min) using a centrifuge (Universal 320R, Hettich, Germany), after which the supernatant was collected for the subsequent analysis. All the operations were performed at 4 °C. Hydrogen peroxide (H_2_O_2_, A064-1-1), total antioxidative capacity (T-AOC, A015-3-1), glutathione (GSH, A006-2-1), glutathione peroxidase (GPX, A005-1-1), and malondialdehyde (MDA, A003-1) levels in the jejunum and liver were quantified using kits (Nanjing Jiancheng Bioengineering Institute, Nanjing, China) according to the manufacturer’s instructions.

### Analysis of the histopathology and ultrastructure

The liver and jejunum samples were washed, paraffin-embedded, dewaxed, sectioned, and stained with hematoxylin-eosin (HE). The stained slides were scanned using a VS120 slide scanner (Olympus, Hamburg, Germany), and three fields of view for each section were randomly selected to be photographed and measured using ZYFviewer (Winmedic, Shandong, China) software. For the liver, three fields in the centrilobular region were randomly selected from each slice to count nuclei (500×).

Glutaraldehyde-fixed 1-mm^3^ liver samples were rinsed, fixed, stained, dehydrated, and cut into ultrathin sections. Next, ultrastructural analysis of liver samples was performed using transmission electron microscopy (Hitachi H-7650, Tokyo, Japan).

### RNA isolation and quantitative real-time PCR

Total RNA was extracted from the liver and jejunum tissues using TRIzol (Takara, 9109, Dalian, China). RNA concentration was measured by measuring the absorbance at 260 nm, and the purity of RNA was examined by measuring the absorbance (A_260_/A_280_) ratio with 1.8–2.0. Total RNA was reverse transcribed using the PrimeScript^TM^ RT reagent kit (Takara, RR047A, Dalian, China). qRT-PCR was performed using Green Premix Ex Ta kit (Takara, RR420A, Dalian, China) in ABI 7500 fast RT-PCR system. The primers used are described in Table S1.

### Western blot analysis

Total protein was lysed by RIPA lysis buffer (Beyotime Biotechnology, P0013B, Shanghai, China) with PMSF (Beyotime Biotechnology, ST506-2, Shanghai, China), and the concentration of total protein in the sample was determined using a BCA protein assay kit (Beyotime Biotechnology, P0010S, Shanghai, China). Briefly, the samples were separated by SDS-PAGE (Beyotime Biotechnology, P0015L, Shanghai, China) and transferred to polyvinylidene fluoride membrane. The membranes were blocked in 5% non-fat dry milk in TBST (2 h, 37 °C), incubated with primary antibodies (12 h, 4 °C), washed 3 times with TBST and incubated with secondary antibodies (1 h, 37 °C). The primary antibodies β-actin (ABclonal, AC026, 1:50,000), carnitine palmitoyltransferase 1 (CPT1A, ABclonal, A20746, 1:1,000), fatty acid transport protein 1 (FATP1, ABclonal, A12847, 1:1,000), adipose triglyceride lipase (ATGL, Wanleibio, WL05654, 1:500), AMPK (ABclonal, A12718, 1:500), AMPK phosphorylation (p-AMPK, ABclonal, AP0432, 1:500), optic atrophy 1 (OPA1, Bioss, bs11764R, 1:1,000), fission 1 (Fis1, Proteintech, 10956-1-AP, 1:1,000), nuclear factor erythroid 2-related factor (Nrf2, ABclonal, A0674, 1:1,000), NAD(P)H quinone oxidoreductase 1 (NQO1, ABclonal, A0047, 1:500), activating transcription factor 4 (ATF4, ABclonal, A18687, 1:500), eukaryotic translation initiation factor 2α (eIF2α, ABclonal, A0764, 1:500), Bcl2-associated X protein (Bax, ABclonal, A19684, 1:1,000), B-cell lymphoma-2 (Bcl-2, Wanleibio, WL01556, 1:500), Caspase-3 (Wanleibio, WL02117, 1:1,000) were used. Protein bands were detected using the enhanced chemiluminescence detection kit (Beyotime Biotechnology, P0018AS, Shanghai, China) and Alpha Imager 2200 (Alpha Innotech Corporation, CA, USA). The proteins concentrations were normalized to that of a β-Actin loading control, and the phospho-protein was normalized to the corresponding total protein concentrations. The ratios of the abundance of normalized phospho-proteins to that of normalized total proteins were calculated and plotted.

### Statistical analysis

SPSS 27.0 (IBM-SPSS Inc, Chicago, IL, USA) and GraphPad Prism 8 (GraphPad Prism Inc., La Jolla, CA, USA) were utilized for statistical analyses and data visualization. One-way ANOVA with Duncan’s multiple comparison test was used for statistical evaluation as indicated. The values are presented as the mean ± SEM, and *P* < 0.05 was considered to indicate statistical significance.

## Results

### Phenotypic characteristics and intestinal nutrient absorption

**Fig. 1:**
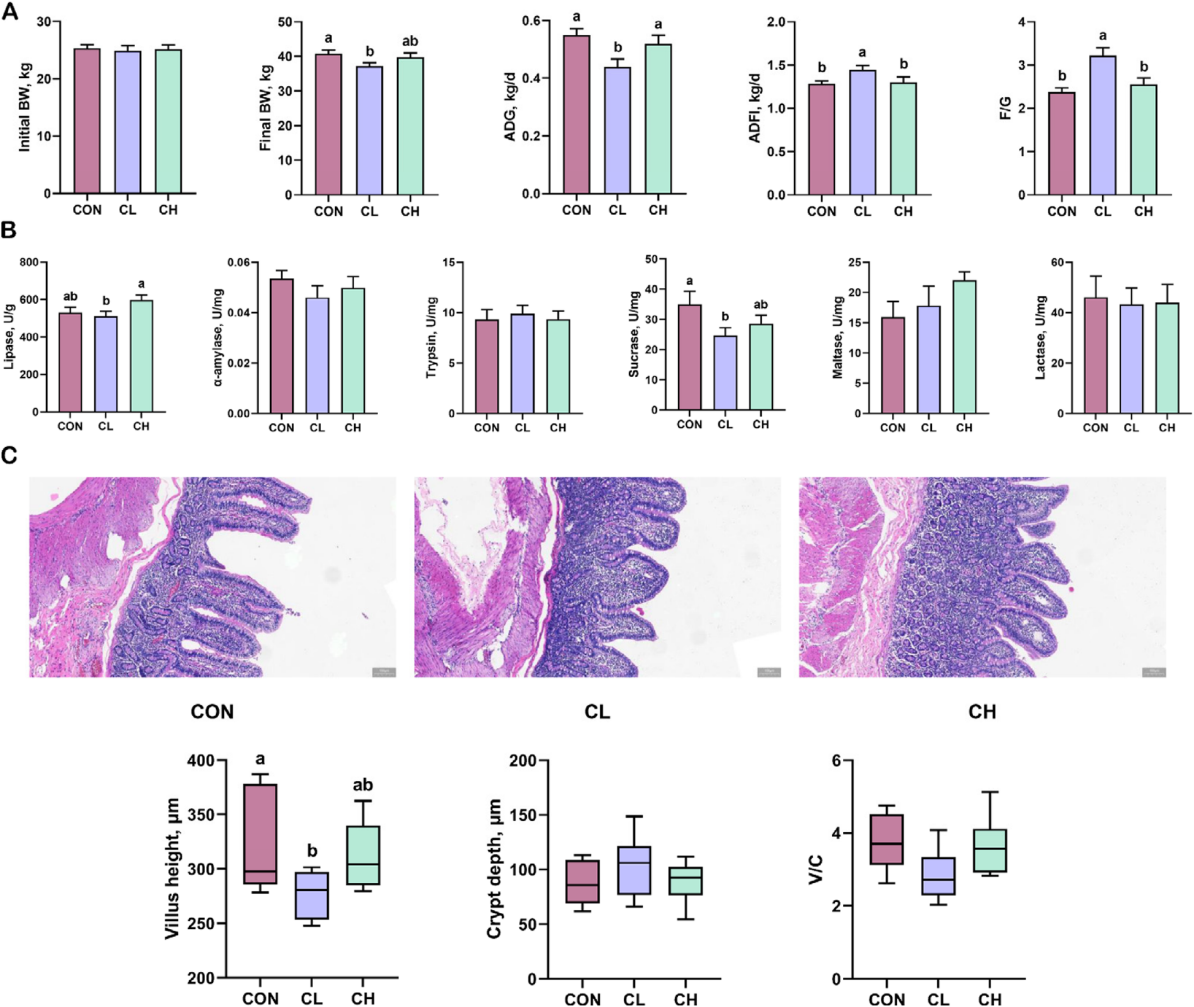
Growth phenotype, intestinal digestive enzymes and morphology in pigs (*n* = 6). **A** Growth performance and feed intake. **B** Activity of digestive enzymes in the jejunal mucosa. **C** Determination of jejunal morphology. ^a,b^Values without the same letters within the same line indicate a significant difference (*P* < 0.05)

The growth performance of the pigs is shown in Fig. 1A. The average daily gain (ADG) was significantly lower in the CL group, and the average daily feed intake (ADFI) and F/G were significantly higher in the CL group than in the CON and CH groups. However, there was no significant difference in growth performance between the CON and CH groups. To investigate the reasons for these phenotypic differences, we determined digestive enzyme activity in the jejunum among the groups (Fig. 1B). Compared with the CL group, the lipase activity in the CH group was significantly increased. At the same time, the sucrase activity in the CL group was significantly lower than that in the CON group, but there was no significant difference in sucrase activity compared with that in the CH group. In addition, no significant differences were observed in the α-amylase, trypsin, maltase, and lactase activities among the experimental treatments. The results of jejunal morphology showed that the villus height was significantly reduced in the CL group, but there was no significant difference compared with the CH group (Fig. 1C).

### Glucose metabolism in the jejunum

**Fig. 2:**
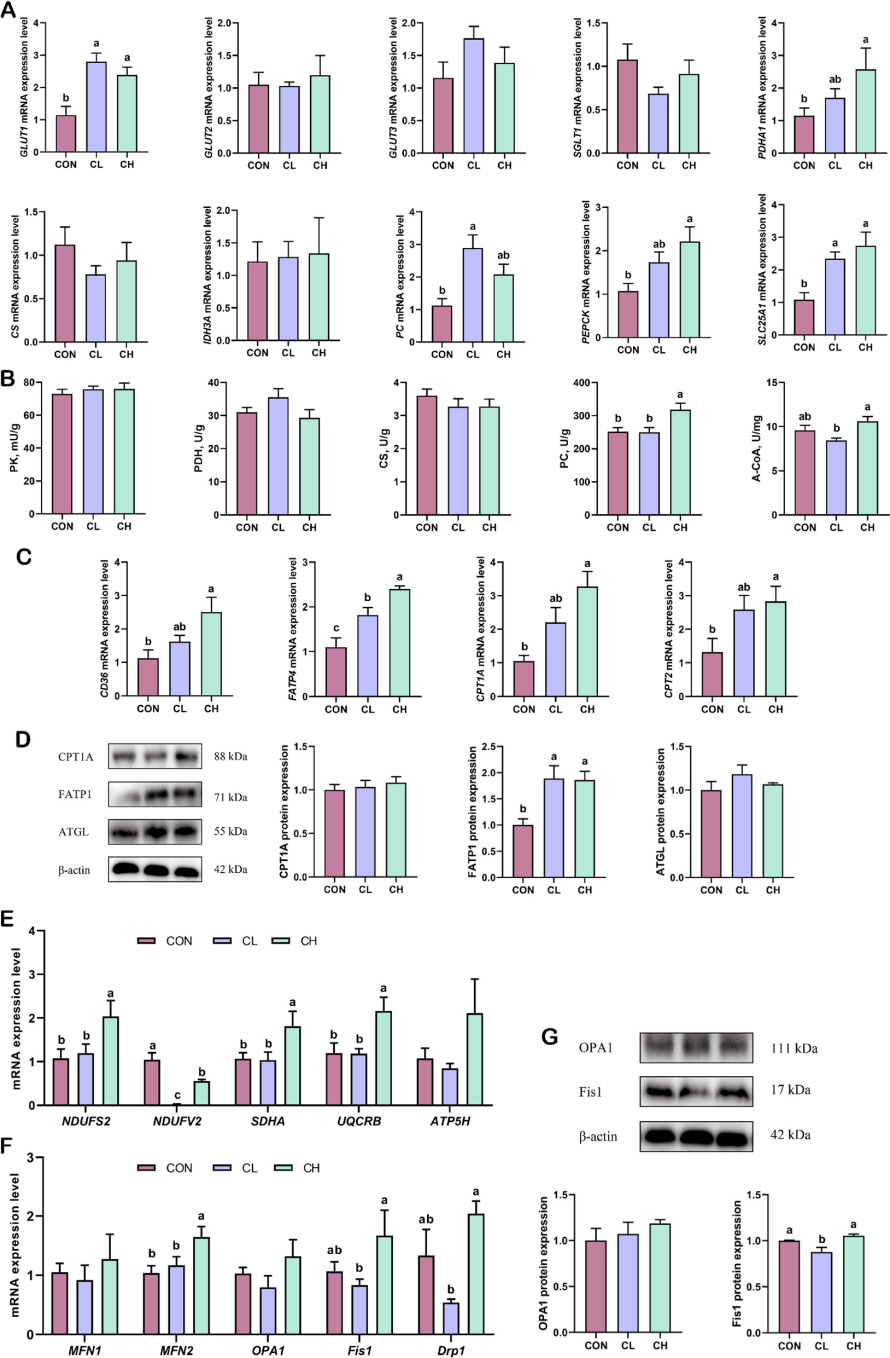
Glycolipid metabolism and mitochondrial function in the jejunum (*n* = 6). **A** Relative mRNA expression of glucose transport and metabolism-related genes. **B** The activity of enzymes related to glucose metabolism. **C** Relative mRNA expression of fatty acid transporters in cell membranes and mitochondrial membranes. **D** Protein expression of fatty acid transporters and adipose triglyceride lipase (*n* = 3). **E** Relative mRNA expression of mitochondrial respiratory chain genes. **F** Relative mRNA expression of mitochondria fusion and division. **G** Protein expression of mitochondria fusion and division (*n* = 3). ^a,b^Values without the same letters within the same line indicate a significant difference (*P* < 0.05)

The jejunum is the main organ responsible for lipid and glucose absorption. Therefore, we assessed the glycolipid metabolism in pigs at the mRNA and protein levels. The relative mRNA expression of various genes related to glucose metabolism are shown in Fig. 2A. Compared with the CON group, the relative mRNA expression of glucose transporter (*GLUT*)1, *PC*, and solute carrier 25A1 (*SLC25A1*) were significantly increased in the CL group, while the expression of *GLUT1*, pyruvate dehydrogenase alpha 1 (*PDHA1*), phosphoenolpyruvate carboxykinase (*PEPCK*), and *SLC25A1* were significantly increased in the CH group. Based on the relative mRNA expression analysis, the glucose-metabolizing enzymes were further validated by ELISA (Fig. 2B). As expected, the activity of PC and A-CoA was higher in the CH group than in the CL group.

### Lipid metabolism in the jejunum

Compared with the CON group, the relative expression of *FATP4* in the CL group was significantly increased, and the relative mRNA expression of fatty acid translocase CD36 (*CD36*), *FATP4*, *CPT1A*, and carnitine palmitoyltransferase 2 (*CPT2*) was significantly increased in the CH group (Fig. 2C). Consistently, the protein expression of FATP1 was significantly increased in both the CL and CH groups, but there was no significant difference in the protein expression of CPT1A and ATGL (Fig. 2D).

### Mitochondrial function in the jejunum

Mitochondria are the energy factories of the cell, and subunits of the complex in the mitochondrial respiratory chain are essential for energy production. As shown in Fig. 2E, the relative mRNA expression of NADH dehydrogenase (ubiquinone) Fe-S protein 2 (*NDUFS2*), NADH dehydrogenase (ubiquinone) flavoprotein 2 (*NDUFV2*), ubiquinol-cytochrome c reductase binding protein (*UQCRB*), and succinate dehydrogenase subunit A (*SDHA*) were significantly increased in the CH group compared with the CL group. Mitochondrial dynamics include fusion and division, which are essential for mitochondrial function (Fig. 2F). The relative mRNA expression of mitofusin (*Mfn*) 2, *Fis1*, and dynamin-related protein 1 (*Drp1*) was significantly higher in the CH group compared with the CL group. Consistently, the protein expression of Fis1 was significantly increased in the CH groups compared with the CL group (Fig. 2G).

### Oxidative stress in the jejunum

**Fig. 3:**
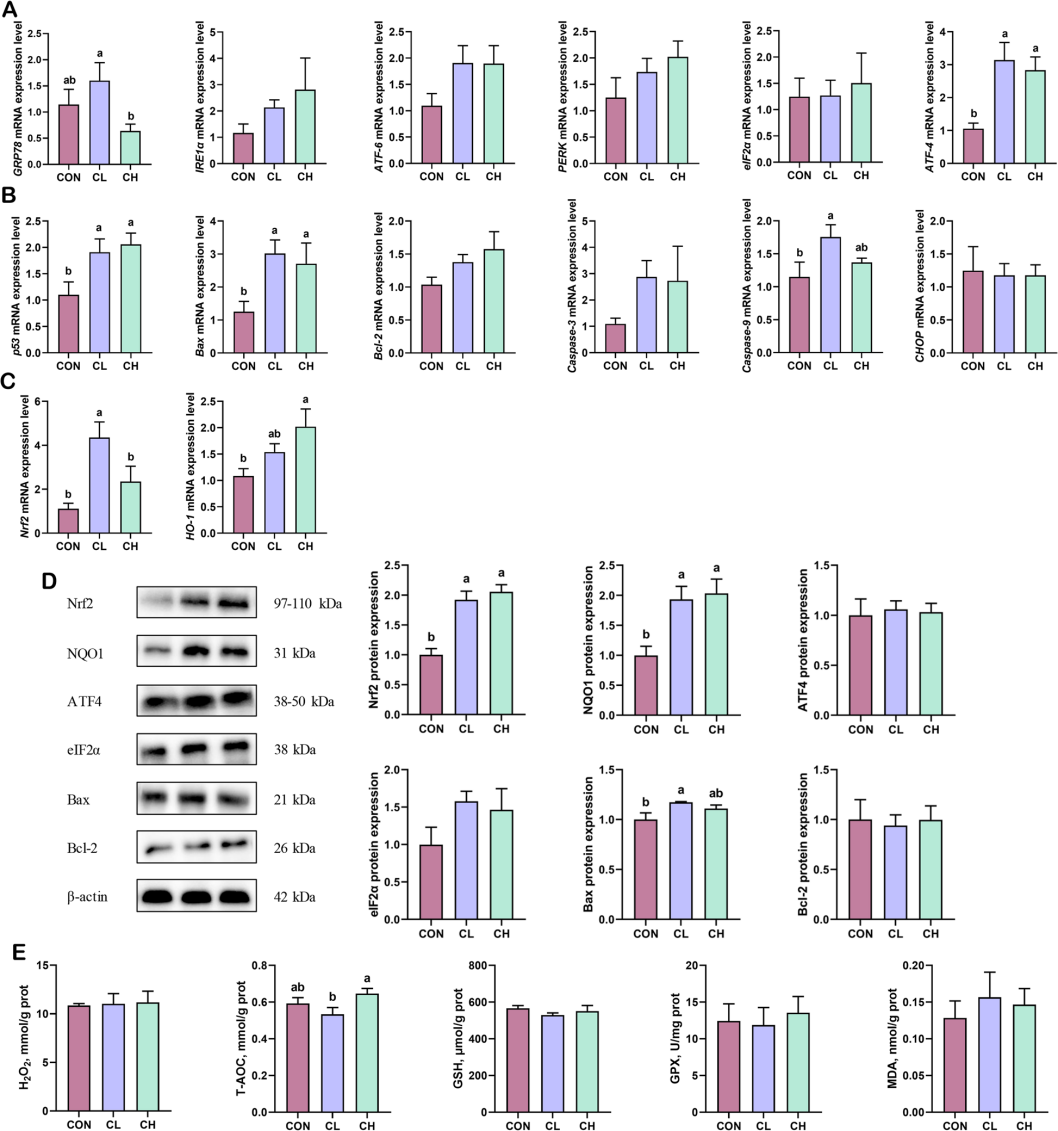
The state of oxidative stress and apoptosis in the jejunum (*n* = 6). **A** Relative mRNA expression of ER stress genes. **B** Relative mRNA expression of apoptotic genes. **C** Relative mRNA expression of the Nrf2-mediated antioxidant systems genes. **D** Protein expression of oxidative stress and apoptosis (*n* = 3). **E** Activity of oxidative stress-related enzymes and markers. ^a,b^Values without the same letters within the same line indicate a significant difference (*P* < 0.05)

As shown in Fig. 3A–C, we found that the mRNA expression of *Nrf2*, *p53*, *Bax*, *Csapase-9*, and *ATF-4* increased significantly in the CL group. In contrast, there were no significant differences in the mRNA expression of *Nrf2* and *Caspase-9* between the CH group and the CON group. For *Nrf2* and glucose-regulated protein 78 (*GRP78*), the pigs fed a high-fat diet had lower relative mRNA expression compared with the CL group. Next, protein expression was evaluated by Western blotting and ELISA. As shown in Fig. 3D, cold temperature exposure significantly increased the protein levels of Nrf2 and NQO1. Meanwhile, the protein expression of Bax in the CL group was significantly lower compared with the CON group, while there was no significant difference in the CL group compared with the CH group. In addition, the level of T-AOC was significantly higher in the CH group than in the CL group, while there was no significant difference in the other markers of oxidative stress (Fig. 3E).

### Metabolite composition in the ileum

**Fig. 4:**
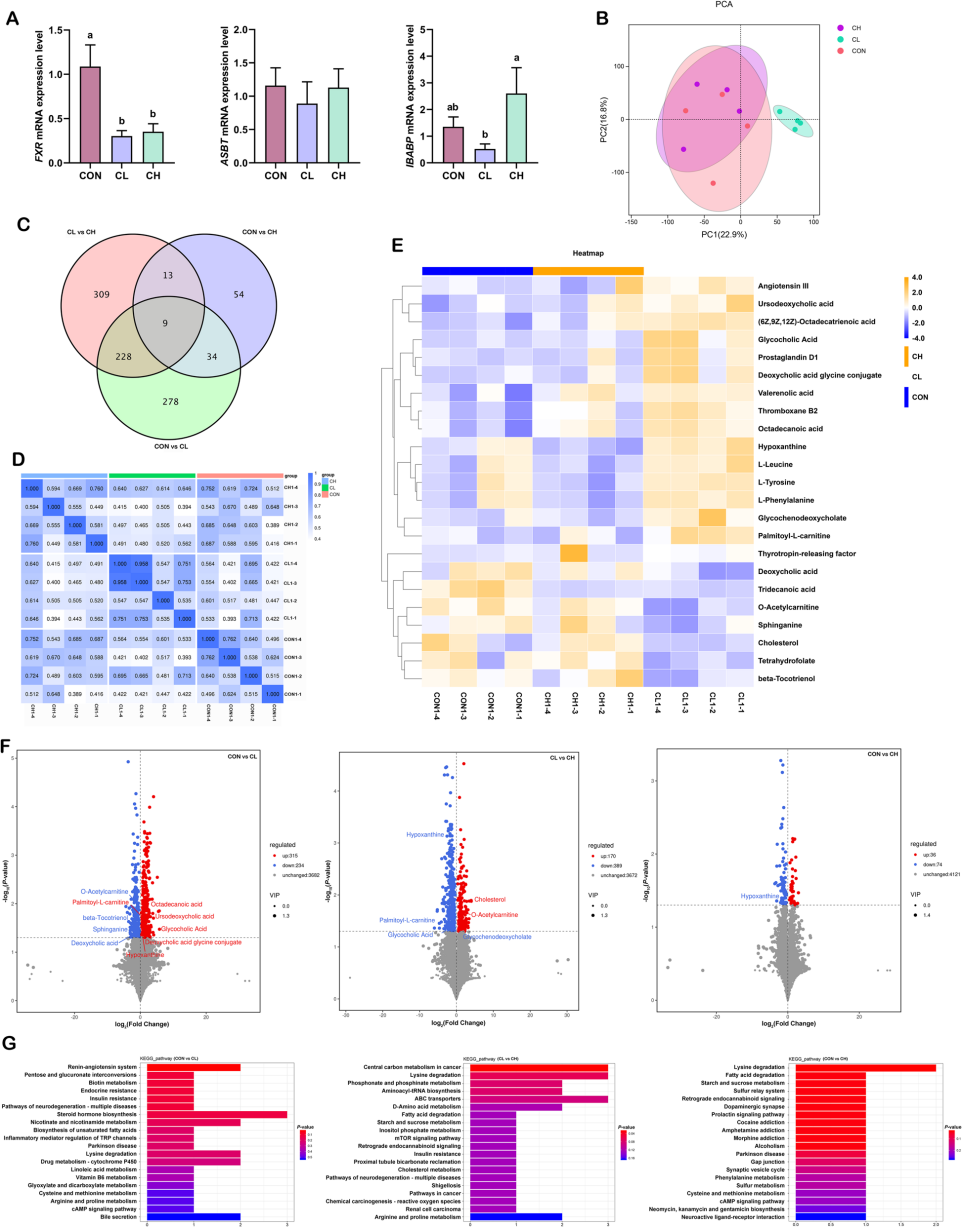
Analysis of composition and differences of metabolites in ileal contents (*n* = 4). **A** Relative mRNA expression of bile acid metabolism-related genes (*n* = 6). **B** Principal component analysis (PCA) of metabolite composition. **C** Venn diagram for differential metabolites in the comparisons CON vs. CL, CL vs. CH, and CON vs. CH. **D** Correlation analysis of samples. **E** Clustering heatmap of the metabolites. **F** Differential metabolites in the comparisons CON vs. CL, CL vs. CH, and CON vs. CH. **G** KEGG pathway of differential metabolites in the comparisons CON vs. CL, CL vs. CH, and CON vs. CH. Red for upregulation and blue for downregulation. ^a,b^Values without the same letters within the same line indicate a significant difference (*P* < 0.05)

As shown in Fig. 4A, the relative expression of ileal bile acid binding protein (*IBABP*) in the CH group was significantly increased compared with the CL group, whereas the expression of farnesoid X receptor (*FXR*) was significantly reduced in both the CL and CH groups. Bile acids and their derivatives facilitate the digestion and absorption of lipids, and to gain further insight into the state of lipid digestion and absorption among groups, an untargeted metabolomics analysis of ileal contents was performed. In the metabolic profiling, 2,244 negative-mode features and 1,988 positive-mode features were identified and subjected to analysis. Precise results were obtained via principal component analysis (PCA), which indicated a significant change in ileal metabolites under cold temperature, whereas metabolic homeostasis was restored after dietary fat supplementation (Fig. 4B and E). The Venn diagram showed the same results as PCA (Fig. 4C). Next, the correlation between samples was determined by Spearman rank correlation analysis. The results revealed stronger correlations within the CL group and weaker correlations with the CON and CH groups, thus verifying that the differential metabolites were reliable and could be subjected to further analyse (Fig. 4D). We analyzed metabolites in the ileum and found 559 differential metabolites between the CON and CL groups, among which 389 metabolites were downregulated and 170 upregulated (Fig. 4F). Next, we carried out KEGG pathway enrichment analysis, and the enriched KEGG pathways (Top 20) were mainly associated with steroid hormone biosynthesis, bile secretion, cAMP signaling pathway, renin-angiotensin system, lysine degradation, and insulin resistance (Fig. 4G). Next, the differential metabolites between the CL and CH groups were compared. A total of 559 metabolites were significantly different in the CH group compared with the CL group, including 170 upregulated and 389 downregulated metabolites (Fig. 4F). Subsequent KEGG pathway analysis (Top 20) showed that these differential metabolites were related to central carbon metabolism in cancer, lysine degradation, ABC transporters, arginine and proline metabolism, and insulin resistance (Fig. 4G). The common pathways enriched in the comparisons of CON vs. CL and CL vs. CH included insulin resistance, bile secretion, steroid hormone biosynthesis, lysine degradation, nicotinate and nicotinamide metabolism, and arginine and proline metabolism. There were only a few differential metabolites were observed between the CON and CH groups; these metabolites were mainly associated with lysine degradation, fatty acid degradation, starch and sucrose metabolism, and neuroactive ligand‒receptor interaction.

### Plasma hormones and biochemical indicators

The portal vein is an important medium that connects the gut-liver axis. For hormones, the concentrations of glucagon and GC were significantly higher in the CL treatment group than in the CON group, and the insulin concentrations were significantly lower (Table 2). Interestingly, no significant differences were observed between the CH and CON groups for the above-mentioned hormones, suggesting that hormonal homeostasis was restored. Furthermore, the levels of TBA and LDL were significantly higher in the CL group compared with the CH group, while there were no significant differences in other biochemical parameters in the plasma (Table 2). The above results suggested that cold temperature exposure induced abnormal lipid metabolism in pigs.

**Table 2:** Effect of dietary fat supplementation on plasma hormones and biochemical indicators in pigs at cold temperatures (*n* = 6)

Items	Treatments			SEM	* P*-value
	CON	CL	CH		
Insulin, mIU/L	39.05^a^	35.22^b^	39.85^a^	0.834	0.038
Glucagon, pg/mL	219.11^b^	266.47^a^	210.28^b^	10.330	0.047
GC, ng/mL	23.23^b^	29.39^a^	27.86^ab^	1.134	0.060
TP, g/L	76.37	73.61	73.20	1.444	0.648
ALB, g/L	24.87	27.23	27.06	0.968	0.559
GLB, g/L	51.50	46.38	46.14	1.677	0.351
GLU, mmol/L	4.85	4.78	4.35	0.233	0.648
TBA, µmol/L	22.66^b^	113.37^a^	39.83^b^	14.340	0.015
urea, µmol/L	4.57	4.55	4.54	0.255	0.999
HDL, mmol/L	0.89	0.84	0.87	0.029	0.758
LDL, mmol/L	1.72^ab^	1.77^a^	1.42^b^	0.068	0.056

*ALB* Albumin, *GC* Glucocorticoid, *GLB* Globulin, *GLU* Glucose, *HDL* High-density lipoprotein, *LDL* Low-density lipoprotein, *TBA* Total bile acid, *TP* Total protein

^a,b^Values without the same letters within the same line indicate a significant difference (*P* < 0.05)

### Glucose metabolism in the liver

**Fig. 5:**
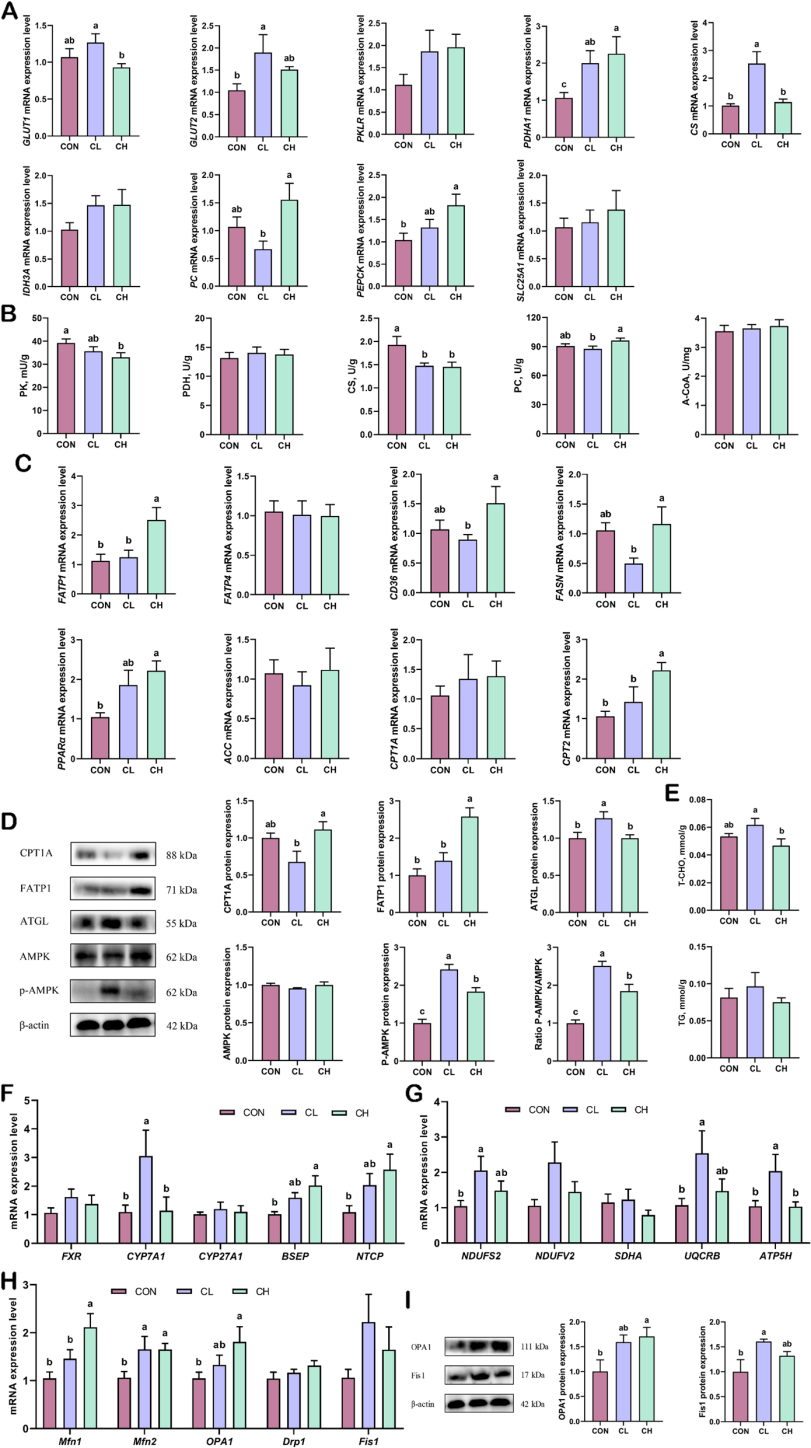
Glycolipid metabolism and mitochondrial function in the liver (*n* = 6). **A** Relative mRNA expression of glucose transport and metabolism-related genes. **B** The activity of enzymes related to glucose metabolism. **C** Relative mRNA expression of fatty acid transporters in cell membranes and mitochondrial membranes. **D** Protein expression of lipid metabolism and AMPK (*n* = 3). **E** T-CHO and TG levels. **F** Relative mRNA expression of bile acid metabolism-related genes. **G** Relative mRNA expression of mitochondrial respiratory chain genes. **H** Relative mRNA expression of mitochondria fusion and division. **I** Protein expression of mitochondria fusion and division (*n* = 3). ^a,b^Values without the same letters within the same line indicate a significant difference (*P* < 0.05)

The relative mRNA expression for various genes related to glucose metabolism are presented in Fig. 5A. Compared with the CON group, the relative mRNA expression of *GLUT2* and *CS* were significantly increased in the CL group, while the protein expression of CS exhibited the opposite trend. Furthermore, dietary fat supplementation upregulated the relative mRNA expression of *PDHA1* and *PEPCK* compared with the CON group. Next, ELISA analysis showed that dietary fat supplementation decreased PK and CS activity, with no significant changes in PDH and A-COA among the groups (Fig. 5B). Notably, dietary fat supplementation at cold temperatures increased the levels of PC (Fig. 5B). These data suggested that cold temperature exposure enhanced glucose transport, and dietary fat supplementation downregulated glucose transport and glycolysis, which may be regulated by hormone secretion.

### Lipid metabolism in the liver

The relative mRNA expression of *FATP1*, fatty acid synthase (*FASN*), *CD36* and *CPT2* were significantly higher in the CH group than in the CL group (Fig. 5C). In addition, the relative mRNA expression of peroxisome proliferator-activated receptor alpha (*PPARα*) was significantly higher in the CH group than in the CON group, but there were no significant changes in the relative mRNA expression of *FATP4*, acetyl-CoA carboxylase (*ACC*), and *CPT1A* among the three groups (Fig. 5C). As shown in Fig. 5D, Western blot analysis showed that cold temperature reduced the protein expression of CPT1A and increased the protein expression of ATGL in the liver. In terms of bile acid metabolism, the relative mRNA expression of cholesterol 7α-hydroxylase (*CYP7A1*) was significantly increased in the CL group (Fig. 5F). In addition, the relative mRNA expression of bile salt export pump (*BSEP*) and sodium taurocholate cotransporting polypeptide (*NTCP*) were significantly increased in the CH group, and there were no significant changes in the *FXR* and sterol 27-hydroxylase (*CYP27A1*) among the three groups (Fig. 5F). Next, we measured the levels of liver T-CHO and TG, and the results showed that T-CHO was significantly higher in the CL group than in the CH group (Fig. 5E). Consistent with the results of plasma TBA measurements, this finding further revealed that cold temperature exposure may lead to abnormal lipid metabolism. Activated AMPK (phosphorylates AMPK at threonine 172) regulates energy metabolism and mitochondrial function. To examine the activation of AMPK in the liver of pigs, Western blot analysis of p-AMPK and AMPK was performed (Fig. 5D). The ratio of p-AMPK to AMPK was significantly increased in the CL group compared to that in the CON and CH groups. Although the ratio of p-AMPK/AMPK was reduced in the CH group, and there was a significant difference compared to the CON group.

### Mitochondrial function in the liver

As shown in Fig. 5G, the relative mRNA expression of *NDUFS2*, *UQCRB*, and ATP synthase subunit d (*ATP5H*) were significantly increased in the CL group compared with that in the CON group, while there were no significant changes in the mRNA expression of *NDUFS2*, *UQCRB*, and *ATP5H* in the CH group. Furthermore, dietary fat supplementation upregulated the relative mRNA expression of *PDHA1* and *PEPCK* compared with the CON group. The mRNA expression of of genes related to mitochondrial fusion and division is shown in Fig. 5H, dietary fat supplementation significantly increased the mRNA expression of *Mfn1*, *Mfn2*, and *OPA1* compared with the CON group. Next, we further validated the protein expression by Western blotting (Fig. 5I). Consistently, the protein expression of OPA1 was significantly increased in the CH group compared to that in the CON group. Notably, a significant increase in the protein expression of Fis1 was observed in the CL group. These data suggested that cold temperature exposure induced mitochondrial dysfunction in the liver, and dietary fat supplementation alleviated mitochondrial dysfunction by promoting mitochondrial fusion.

### Histopathology and ultrastructure in the liver

**Fig. 6:**
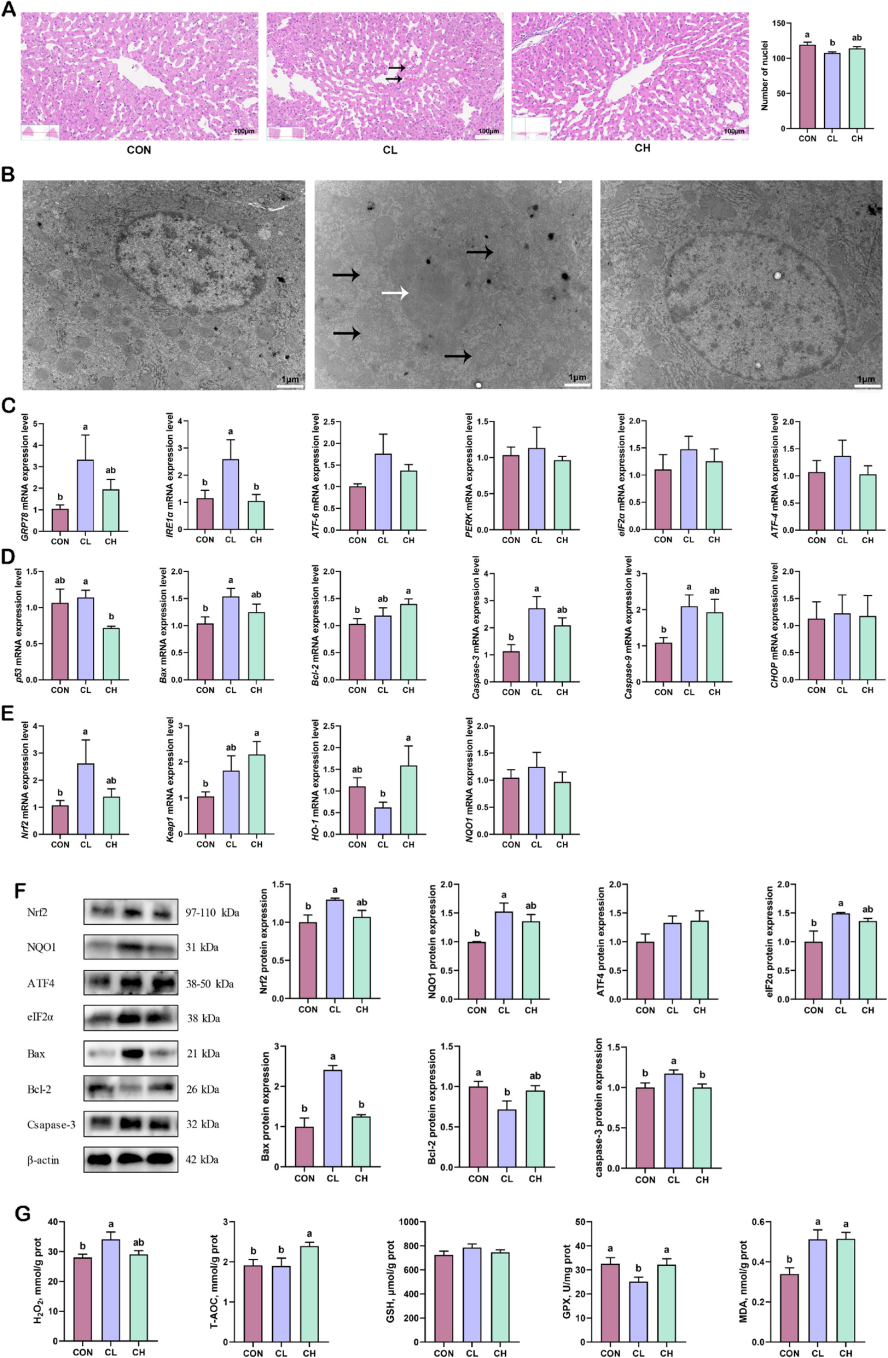
The state of oxidative stress and apoptosis in the liver (*n* = 6). **A** HE staining of liver (Black arrows indicate hemorrhagic spots), magnification 200×. **B** Ultrastructural analysis of liver (Black arrows indicate mitochondrial swelling, white arrows indicate abnormal nuclear morphology), magnification 15,000×. **C** Relative mRNA expression of ER stress genes. **D** Relative mRNA expression of apoptotic genes. **E** Relative mRNA expression of the Nrf2-mediated antioxidant systems genes. **F** Protein expression of oxidative stress and apoptosis (*n* = 3). **G** Activity of oxidative stress-related enzymes and markers. ^a,b^Values without the same letters within the same line indicate a significant difference (*P* < 0.05)

Histological examination showed vacuolization of hepatocytes in the CL group, a decrease in the number of nuclei in the centrilobular region, and the presence of hemorrhagic spots between the hepatic sinusoids (Fig. 6A). In addition, mild vacuolization occurred in the CH group, but no decrease in the number of nuclei and obvious hemorrhagic spots were observed (Fig. 6A).

Next, we observed ultrastructural changes in the liver (Fig. 6B). Cold temperature induced mitochondrial swelling, rounding, partial cristae dissolution, and mitochondrial and cytoplasmic vacuolization. At the same time, the morphological abnormalities in the nucleus mainly manifested as spacing dilation. In addition, we found an increase in the number of mitochondrial fusions, ER, ribosomes, and nuclear pores in the CH group, suggesting more vigorous protein synthesis and metabolism in the cells. To our surprise, no morphological abnormalities in the nucleus and ER were observed, and only slight mitochondrial cristae dissolution was observed.

### Oxidative stress in the liver

An increase in ROS may induce apoptosis by increasing oxidative stress damage in the liver. As shown in Fig. 6C–E, the relative mRNA expression of *Nrf2*, *Bax*, *Caspase-3*, *Caspase-9*, *GPR78*, and inositol-requiring enzyme 1α (*IRE1α*) were significantly increased in the CL group, while there were no significant changes in the CH group. In addition, dietary fat supplementation upregulated the relative mRNA expression of kelch-like ECH-associated protein 1 (*Keap1*) and *Bcl-2* compared with the CON group. Consistent with these results, Western blotting showed that cold temperature exposure increased Nrf2, NQO1, eIF2α, Bax, and Caspase-3 protein expression, and decreased Bcl-2 protein expression compared with the CON group (Fig. 6F). In addition, no significant differences were observed in the protein expression of Nrf2, NQO1, eIF2α, Bax, Bcl-2, and Caspase-3 in the CH group compared with the CON group (Fig. 6F). Next, we determined the levels of oxidative stress markers by ELISA. As shown in Fig. 6G, cold temperature exposure increased H_2_O_2_ and MDA levels, and decreased GPX levels. Furthermore, the levels of T-AOC and MDA were significantly increased in the CH group compared to that in the CON group, with no significant changes in the levels of H_2_O_2_, GSH, and GPX. Overall, we found that dietary fat supplementation alleviated oxidative stress and apoptosis in the liver to some extent, which may be a result of the alleviation of energy stress.

## Discussion

The absence of brown fat reservoirs in pigs increases sensitivity to cold temperature, resulting in disease, stunted growth, and even death [34]. Farming in cold temperate zones increases the cost of livestock production and reduces production efficiency, so strengthening the sustainable development of animal husbandry is essential for addressing climate challenge. Pigs tend to cope with cold stress by increasing energy intake, which is consistent with our findings [7, 35]. Excitingly, dietary fat supplementation alleviated the negative effects of cold stress on piglet performance. Glucose is first consumed when the host is in a cold environment, and hormones are mobilized to regulate energy metabolism as cold temperature exposure is prolonged [36]. Consistently, we did not observe changes in blood glucose levels in cold temperature exposure; instead, we observed changes in the levels of hormones that regulate metabolic homeostasis. The liver is the target organ of insulin, glucagon, and glucocorticoids, and the hormonal changes observed in this study may induce an increase in glucose output by the liver [37]. In addition, higher TBA and LDL levels were observed in the CL group, which suggested impaired lipid metabolism in pigs. Abnormalities in lipid metabolism may lead to insulin resistance and excessive glucose production, so we further evaluated glycolipid metabolism status in pigs [38].

Nutrients are absorbed through the intestine and transferred to the liver, and the blood is the bridge between the intestine and the liver, so we evaluated the energy metabolism of pigs via the gut-liver axis [39]. Surprisingly, among the digestive enzymes, the results showed that cold stress only reduced jejunal sucrase activity. However, it should be pointed out that dietary fat supplementation increased lipase activity and improved intestinal villi damage at cold temperatures. Therefore, it seems plausible to infer that dietary fat supplementation increases glucose and fatty acid transport and absorption, but the regulatory mechanisms remain unclear. Bile acids and their derivatives facilitate the digestion and absorption of lipids, and more than 95% of the bile acids secreted by the liver are reabsorbed at the end of the ileum to complete enterohepatic circulation. Metabolomics showed that the metabolite composition in the CON group was similar to that in the CH group, but with higher variation than that of the CL group. These results suggested abnormalities in lipid metabolism in the CL group, which was characterized by the accumulation of glycochenodeoxycholate, glycocholic acid, deoxycholic acid glycine conjugate, ursodeoxycholic acid, and palmitoyl-L-carnitine. Previous studies have reported similar results that cold stress increases the levels of glycocholic acid and glycochenodeoxycholate [36]. Consistently, these differential metabolites are primarily enriched in bile secretion, steroid hormone biosynthesis, and insulin resistance, which are closely related to energy metabolism. Interestingly, we observed down-regulation of glycochenodeoxycholate, glycocholic acid, and palmitoyl-L-carnitine and up-regulation of o-acetylcarnitine and cholesterol in the CH group, which revealed enhanced lipid metabolism. A recent study reported that o-acetylcarnitine deficiency indicates impaired mitochondrial function, so dietary fat supplementation has the potential to restore mitochondrial function at cold temperatures [40]. We speculate that growth inhibition at cold temperatures may be due to glycolipid metabolism dysregulation, which leads to energy stress and mitochondrial damage in pigs.

Next, we further determined the transcriptional and translational regulation of glycolipid metabolism and oxidative stress in the jejunum and liver. The mRNA expression of jejunal *GLUT1* increased significantly at cold temperatures, suggesting that cold temperature increased jejunal glucose transport. Interestingly, we did not observe a great change in jejunal glucose metabolism status in the CL group. However, the rate of jejunal glucose metabolism in the CH group increased significantly, which was characterized by an enhanced tricarboxylic acid (TCA) cycle. A-CoA is the central metabolite linking glycolysis, TCA cycle, lipid biosynthesis, fatty acid oxidation, and ketogenesis, so glucose metabolism and lipid metabolism are interdependent and interconverted to jointly regulate energy balance [41]. An increase in jejunal A-CoA may indicate an increase in lipid metabolism, a conjecture that was validated by further results. With respect to lipid metabolism, we found that the expression of lipid transport proteins and *IBABP* were higher in the CH group. Mitochondria produce energy primarily through fatty acid oxidation, TCA cycle, and the respiratory chain, while *NDUFS2*, *NDUFV2*, *SDHA*, *UQCRB*, and *ATP5H* are subunits of the mitochondrial respiratory chain complex [42, 43]. Consistently, we found higher expression of multiple subunits of the mitochondrial respiratory chain complex. The enhancement of the mitochondrial respiratory chain may damage mitochondria by inducing ROS production to cause oxidative stress and apoptosis, and mitochondrial fusion and fission are essential for maintaining mitochondrial function [44–46]. We found that mitochondrial fusion and division were in dynamic equilibrium in the CH group, and mitochondria were more active, which contributed to the energy balance and scavenging of ROS. Consistently, no changes were observed in the levels of H_2_O_2_ and MDA in the CH group. Further determination of oxidative stress in the jejunum showed that cold stress activated Nrf2 to maintain redox homeostasis. Moreover, cold stress upregulated the expression of Bax by activating p53, thus further inducing apoptosis. Collectively, although the Nrf2 antioxidant pathway was activated, cold stress also induced apoptosis, and dietary fat supplementation neither induced nor alleviated oxidative stress in the jejunum.

The liver is the core organ that regulates mammalian energy metabolism and can determine the fate of substrates according to the external environment and nutrient availability [12]. Therefore, the status of glycolipid metabolism and oxidative stress in the liver may determine the ultimate fate of the host. We found that cold stress increased glucose transport in the liver, which fully validates the idea that hormones can act on the liver to regulate glucose output. In addition, this result was not observed in the CH group, suggesting that dietary fat supplementation may compensate for energy deficiency by increasing lipid metabolism. PK is a rate-limiting enzyme of the glycolytic pathway, which converts phosphoenopyruvate to pyruvate, while PC and PEPCK are rate-limiting enzymes in the gluconeogenesis pathway. In the CH group, we observed lower expression of PK and higher expression of PC and PEPCK, suggesting that pigs fed a high-fat diet at cold temperature inhibited glycolysis activity and promoted gluconeogenesis activity in the liver. In agreement with our results, previous studies have shown similar results [47]. The increase in fatty acid intake is accompanied by an increase in A-CoA, which leads to pyruvate accumulation to promote gluconeogenesis [48]. In addition, oxaloacetate, glycerol, and ketone bodies are all raw materials for gluconeogenesis, which may account for the increase in gluconeogenesis in the liver of the CH group.

The altered state of glucose metabolism in the CH group is inextricably linked to lipid metabolism, so we further investigated lipid metabolism in the liver. As expected, lipid transport and metabolism were significantly enhanced in the CH group, which was characterized by a significant increase in the expression of *FATP1*, *CD36*, *FASN*, *CPT1A*, and *CPT2* compared to the CL group. Fatty acid uptake requires the involvement of fatty acid transporters, including a family of fatty acid transport proteins and fatty acid translocase [49]. In addition, *CPT1A* and *CPT2* are present in the outer and inner mitochondrial membranes, respectively, and are jointly involved in the beta-oxidation of fatty acids to generate ATP for the host [50]. Excitingly, we also observed a decrease in ATGL expression and an increase in *BSEP* and *NTCP* expression in the CH group. As a rate-limiting enzyme in intracellular triglyceride hydrolysis, ATGL is essential for cold adaptation in pigs, and these results suggest that dietary fat supplementation reduces lipid hydrolysis and increases bile acid recycling to alleviate the negative effects of cold stress on pig growth [51]. Furthermore, even though cold temperature increased bile acid synthesis by increasing the expression of *CYP7A1*, this did not increase lipid metabolism in the liver. Previous studies have shown that the dysregulation of bile acid homeostasis can cause cholestatic liver disease and ER stress, and these results are consistent with our findings [52]. Excitingly, the status of lipid metabolism in the CH group was consistent with the growth performance, while the energy stress due to insufficient ATP production in the CL group may be one of the reasons for the decrease in the growth performance of pigs. AMPK is an energy sensor and regulator of eukaryotic cells that can regulate glycolipid metabolism during starvation by phosphorylation, so we hypothesized that dietary fat supplementation at cold temperatures could alleviate energy stress in pigs by decreasing the phosphorylation of AMPK [14, 15]. We found that the expression of p-AMPK was increased in the CL group, whereas the expression of p-AMPK in the CH group was decreased compared to the CL group, and these results validated our speculation. However, although there was no difference in the growth performance of pigs in the CH group compared with the control group, there was still a difference in the expression of p-AMPK, which may be a basal difference due to cold stress-induced alteration of metabolic status. In addition to orchestrating glucose and lipid metabolism, AMPK is associated with oxidative damage, inflammation, apoptosis, and mitochondrial homeostasis [53, 54]. Further analysis of the state of the respiratory chain and dynamics of mitochondria showed that the level of mitochondrial respiration and division was higher in the CL group, while mitochondrial fusion was not significantly increased. An imbalance in mitochondrial fusion and division disrupts mitochondrial morphology and leads to mitochondrial dysfunction, as well as oxidative stress damage to the liver [55].

**Fig. 7:**
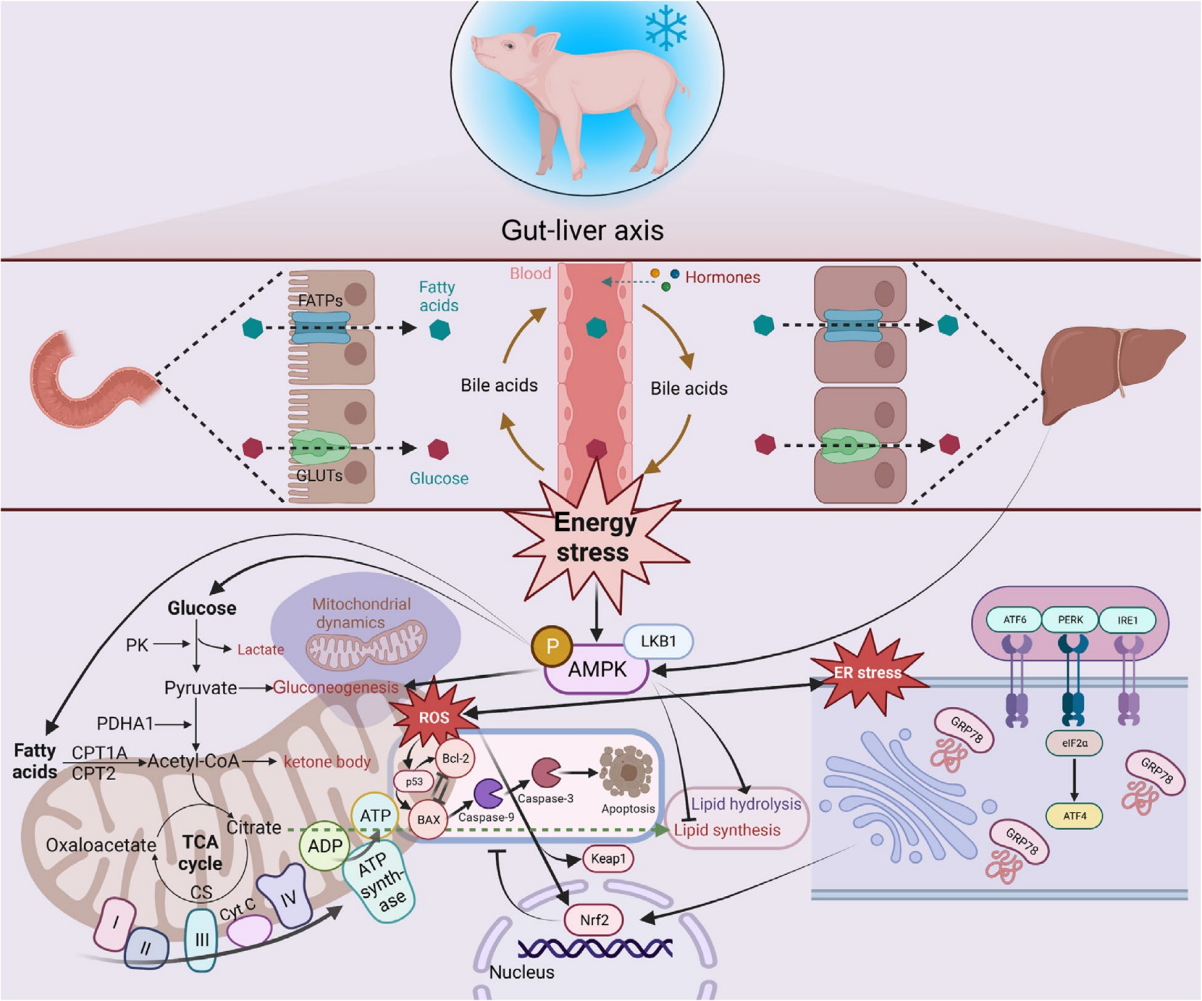
Schematic model illustrating the mechanism of dietary fat supplementation alleviates environmental cold temperature-induced energy stress through AMPK-mediated mitochondrial homeostasis of pigs

Based on the above results, we evaluated the morphology and ultrastructure of the liver. Consistently, cold stress induced vacuolization of liver cells and a decrease in the number of nuclei, which reflects an increase in apoptosis. The increase in apoptosis levels may be induced by mitochondrial damage, and further ultrastructural analysis of the liver revealed abnormalities in mitochondrial morphology and structure. In addition, morphological abnormalities of the nucleus mainly manifested as spacing dilation, suggesting that cold stress caused severe damage to the liver. Excitingly, no significant mitochondrial ultrastructure damage was observed in the CH group, and there was increased protein synthesis and metabolism, which may be because dietary fat supplementation alleviated the energy stress in pigs by reducing the expression of p-AMPK. Sustained activation of AMPK induces apoptosis and oxidative stress, so we further verified the oxidative stress and apoptosis levels in the liver by investigating the mRNA and protein expression. In organisms, a complete oxidative defence system, including antioxidant enzymes and non-enzymatic systems, helps maintain low levels of ROS to reduce oxidative stress. In this study, we found that cold stress increased the levels of H_2_O_2_ and MDA compared with the CON group, while activation of the Nrf2 antioxidant system was also observed. This suggests that cold stress disrupts the ROS balance, which activates the Nrf2-mediated antioxidant defence system to remove the damaged organelles. The ER is also a producer of ROS, and excessive ROS levels can cause ER stress, thus further inducing apoptosis [56]. GRP78, Bax, Bcl-2, and Caspase-3 are markers of ER stress and apoptosis, respectively, where Caspase-3 is the executor and can be activated by Caspase-9 to induce apoptosis [57, 58]. In this study, cold stress induced ER stress and apoptosis in the liver, which is consistent with the results on p-AMPK and mitochondrial function. Excitingly, we observed alleviation of oxidative stress in the CH group, which was characterized by an increase in T-AOC and GPX levels. Endogenous antioxidant enzymes, including GPX, superoxide dismutase, and catalase, provide biological defence against ROS-induced cellular or organ damage [59]. This confirms the critical role of dietary fat supplementation in alleviating cold temperature-induced oxidative stress and damage. The mitochondrial oxidative stress pathway and the ER stress pathway mediate apoptosis simultaneously, and the alleviation of oxidative stress may indicate a decrease in apoptosis [60, 61]. Therefore, we measured the expression of apoptosis-related genes and proteins in the liver. The expression of *p53* was positively correlated with apoptosis, which is consistent with the expression of apoptosis-related proteins in this study. As an energy sensor and regulatory region, AMPK controls cellular metabolism homeostasis by regulating energy production [14, 15]. Prolonged energy stress induces AMPK activation and ROS production, which are directly associated with mitochondrial dysfunction and ER stress [14, 62]. In short, this study demonstrated that dietary fat supplementation at cold temperature improved pig growth by down-regulating p-AMPK expression to alleviate energy stress (Fig. 7). In addition, based on the results of this study on dietary fat supplementation to alleviate energy stress in pigs, we estimate that the use of other fat sources, such as animal fat, will also alleviate the negative effects of cold temperatures on pig growth performance. However, the results of this study are limited to soybean oil as a fat source and are not representative of other fat sources.

## Conclusion

Pigs exposed to cold temperatures showed lower growth performance, abnormal glycolipid metabolism and oxidative stress damage based on the gut-liver axis. Excitingly, pigs fed a high-fat diet showed improved growth performance compared to the CL group. Concomitantly, based on AMPK, a key regulator of energy homeostasis, the pigs showed altered glycolipid metabolism and the reversal of organelle damage caused by oxidative stress. Thus, AMPK-mediated mitochondrial biogenesis and homeostasis in the liver altered energy metabolism to reverse oxidative stress damage. In conclusion, our results show the importance of AMPK-mediated energy homeostasis in pig growth and development at cold temperature and provide a new nutritional strategy for feeding pigs in high-cold zones and seasonal environments.

### Supplementary Information

**Additional file 1: Table S1** The qRT-PCR primer sequences used in this study.

## Data Availability

The datasets produced and/or analyzed during the current study are available from the corresponding author on reasonable request.

## Abbreviations

ACC: Acetyl CoA carboxylase
A-CoA: Acetyl-coenzyme A
ADFI: Average daily feed intake
ADG: Average daily gain
ALB: Albumin
AMPK: AMP-activated protein kinase
ASBT: Apical sodium-dependent bile acid transporter
ATF4: Activating transcription factor 4
ATF6: Activating transcription factor 6
ATP5H: ATP synthase subunit d
Bax: Bcl2-associated X protein
Bcl-2: B-cell lymphoma-2
BSEP: Bile salt export pump
CPT1A: Carnitine palmitoyltransferase 1
CPT2: Carnitine palmitoyltransferase 2
CS: Citrate synthase
CYP7A1: Cholesterol 7α-hydroxylase
CYP27A1: Sterol 27-hydroxylase
Drp1: Dynamin-related protein 1
ER: Endoplasmic reticulum
eIF2α: Eukaryotic translation initiation factor 2α
FASN: Fatty acid synthase
FATP: Fatty acid transport protein
Fis1: Fission 1
FXR: Farnesoid X receptor
GC: Glucocorticoid
GLB: Globulin
GLO: Globulin
GLU: Glucose
GLUT: Glucose transporter
GPX: Glutathione peroxidase
GRP78: Glucose-regulated protein 78
GSH: Glutathione
HDL: High-density lipoprotein
HE: Hematoxylin-eosin
HO-1: Heme oxygenase 1
H_2_O_2_: Hydrogen peroxide
IBABP: Ileal bile acid binding protein
IDH3A: Isocitrate dehydrogenase 3
IRE1α: Inositol-requiring enzyme 1α
Keap1: Kelch-like ECH-associated protein 1
LDL: Low-density lipoprotein
MDA: Malondialdehyde
Mfn: Mitofusin
NDUFS2: NADH dehydrogenase (ubiquinone) Fe-S protein 2
NDUFV2: NADH dehydrogenase (ubiquinone) flavoprotein 2
NQO1: NAD(P)H quinone oxidoreductase 1
Nrf2: Nuclear factor erythroid 2-related factor
NTCP: Sodium taurocholate cotransporting polypeptide
OPA1: Optic atrophy 1
p-AMPK: AMPK phosphorylation
PC: Pyruvate carboxylase
PDH: Pyruvate dehydrogenase
PDHA1: Pyruvate dehydrogenase alpha 1
PEPCK: Phosphoenolpyruvate carboxykinase
PERK: Protein kinase R-like endoplasmic reticulum kinase
PK: Pyruvate kinase
PPARα: Peroxisome proliferator-activated receptor alpha
PUFAs: Polyunsaturated fatty acids
ROS: Reactive oxygen species
SDHA: Succinate dehydrogenase subunit A
SGLT1: Recombinant sodium/glucose cotransporter 1
SLC25A1: Solute carrier 25A1
T-AOC: Total antioxidative capacity
TBA: Total bile acid
TCA: Tricarboxylic acid
T-CHO: Total cholesterol
TG: Triglycerides
TP: Total protein
UQCRB: Ubiquinol-cytochrome c reductase binding protein
