# Supplementary material for: Dietary fat supplementation relieves cold temperature-induced energy stress through AMPK-mediated mitochondrial homeostasis in pigs
Source: J Anim Sci Biotechnol. 2024 Apr 8;15:56. doi: 10.1186/s40104-024-01014-7 (PMC11000307; doi:10.1186/s40104-024-01014-7)
Supplement: Supplementary file 1 — Additional file 1: Table S1 The qRT-PCR primer sequences used in this study. [file 40104_2024_1014_MOESM1_ESM.docx]

Table S1 The qRT-PCR primer sequences used in this study

| Gene | Primer sequence (5’-3’) | Accession number |
| --- | --- | --- |
| *β-actin* | F: GGCACCACACCTTCTACAACGAG | XM_003124280.5 |
|  | R: TCATCTTCTCACGGTTGGCTTTGG |  |
| *CPT1A* | F: AGACACCATCCAGCACCTCCAG | NM_001129805.1 |
|  | R: CAACAGCCTGCCGTCGTAATAGAG |  |
| *CPT2* | F: GAGTGGACGCTGCTGTGTTCTG | NM_001246243.1 |
|  | R: CGGCAAGGATGAGGTTGAAGGAC |  |
| *Caspase-3* | F: AGAATTGGACTGTGGGATTGAGACG | NM_214131.1 |
|  | R: TTTCGCCAGGAATAGTAACCAGGTG |  |
| *Caspase-9* | F: CATTGAGACCCTGGATGGCGTTC | XM_013998997.2 |
|  | R: CCCTTTCACTGAGACAGCATTGGAG |  |
| *Bax* | F: TTTGCTTCAGGGTTTCATCC | XM_ 003127290.3 |
|  | R: GACACTCGCTCAACTTCTTGG |  |
| *Bcl-2* | F: GCGACTTTGCCGAGATGT | XM_021099602.1 |
|  | R: CACAATCCTCCCCCAGTTC |  |
| *P53* | F: GCCCATCCTCACCATCATCACAC | NM_213824.3 |
|  | R: GCACAAACACGCACCTCAAAGC |  |
| *CHOP* | F: GTCATTGCCTTTCTCCTTCGG | NM_001144845.1 |
|  | R: GGTTTTTGACTCCTCCTCATTTCC |  |
| *PKLR* | F: TCCCACTGAAGTCACCGCTATCG | XM_021089721.1 |
|  | R: ACACAATGATGGCAGCAGCACAG |  |
| *IDH3A* | F: GTGACACCAAGCGGCAACATTG | XM_001927338.5 |
|  | R: CATCATCACAGCACTGAGCAGGAG |  |
| *PDHA1* | F: GGCATAAACCCTACGGACCATCTG | XM_003360244.4 |
|  | R: ACCTCCTCTTCGTCCTGTAAGTTCC |  |
| *CS* | F: GCATGACGGAGATGAACTACTACAC | XM_021091143.1 |
|  | R: CCTAAGGCTCGGCTCCAGATG |  |
| *SLC25A1* | F: GTGCGGGAACAAGGGCTGAAG | XM_021071932.1 |
|  | R: CGGTCATAACGAAGAAGCGGATGG |  |
| *PC* | F: GTCTGGATAACGCATCTGCCTTC | NM_214349.1 |
|  | R: CGCCTCGGACTCGGAACTC |  |
| *PEPCK* | F: CTGTGCCAGCCTGACCAAATCC | XM_005673043.3 |
|  | R: AGCCTCTTGATGACACCCTCTTCC |  |
| *GLUT1* | F: ACGGTGCTCCTGGTCCTGTTC | XM_021096908.1 |
|  | R: CTCGGGTGTCTTGTCGCTTTGG |  |
| *GLUT2* | F: TGCTCTGGTCTCTGTCTGTGTCC | NM_001097417.1 |
|  | R: ATTCTTCCAAGCCGATCTCCAAGC |  |
| *GLUT3* | F: CCTGGGCCGATTGGTTATTG | XM_021092392.1 |
|  | R: CAGAATCCCGATGACGATGC |  |
| *SGLT1* | F: TCATCATCGTCCTGGTCGTCTCC | NM_001164021.1 |
|  | R: TGAATGTCCTCCTCCTCTGCATCC |  |
| *FATP1* | F: GTGCTGAGTCGCCTGCTTGG | XM_021076151.1 |
|  | R: CCATGCCTGCTTTGCCCTCTAC |  |
| *FATP4* | F: CTACCACACGGCAGGCAACATC | XM_013993903.2 |
|  | R: ACACAATCATCCCAGAACCGAGAAG |  |
| *CD36* | F: TACAGCCCAATGGTGCCATCTTTG | XM_021102279.1 |
|  | R: TGCCACAGCCAGATTGAGAACAG |  |
| *FASN* | F: ACACCTTCGTGCTGGCCTAC | NM_001099930.1 |
|  | R: ATGTCGGTGAACTGCTGCAC |  |
| *ACC* | F: CAAAGAGGTTCCAGGCACAGTCC | NM_001114269.1 |
|  | R: CGTCAGCATGTCAGAAGGCAGAG |  |
| *PPAR-α* | F: AGCAATAACCCGCCTTTCGTCATAC | NM_001044526.1 |
|  | R: ACCTCCGCCTCCTTGTTCTGG |  |
| *FXR* | F:AAGGACCGAGAGGCAGTAGAGAAG | NM_001287412.1 |
|  | R:GACCCAGGAGGCAGGCAAAATG |  |
| *CYP7A1* | F:TCCCTTGTCCTACCATAAAGTGTTGTG | NM_001005352.3 |
|  | R:GTCAATGCTTCTGTGCCCAAATGC |  |
| *CYP27A1* | F:ACTCACTCTACGCCACCTTCCTC | NM_001243304.1 |
|  | R:GTATTCCAGCCATCCAGGTATCGC |  |
| *BSEP* | F:GAGGTCGCCGCACAGATTACTAATG | XM_003133457.5 |
|  | R:GGATGGCTGTCTTGTATGGCTTCTC |  |
| *BTCP* | F:CAACTTCACCCTCCCGCACAAC | XM_001927695.5 |
|  | R:ATGAGCAGCATGAACACCAGGATG |  |
| *ASBT* | F:TCGCTCTTATTGTTCCTGTGTCCATC | NM_001244463.1 |
|  | R:TCTGGTATAGCACTCCTCCGATCAC |  |
| *IBABP* | F: GCAAGGAGTGCGACATAGAGAC | NM_214215.2 |
|  | R: TGGTGGTAGTTGGGGCTGTT |  |
| *NDUFS2* | F: GAAATGCGCCAGTCCCTTCG | XM_005663167.3 |
|  | R: TGCGCGTTTAGGTGGAGACA |  |
| *NDUFV2* | F: TGAACTCAAGGCTGGCAAAATCCC | NM_001097475.2 |
|  | R: CACACCAAACCCAGGTCCTTTAGG |  |
| *SDHA* | F: ATGGAGGAGGACAACTGGAGGTG | XM_021076931.1 |
|  | R: GCCTGCTCTGTCATGTAGTGGATG |  |
| *UQCRB* | F: CCTGCTGGCTTAATGCGAGATGATAC | XM_021088626.1 |
|  | R: GTCAGGTCCAGTGCTCTCTTAATGC |  |
| *ATP5H* | F: TACCCCGAAACCAGAAGGCC | XM_021066094.1 |
|  | R: TACCAAGCCAGCCTTTGCCA |  |
| *Mfn1* | F: TGGACTTTATCCGAAACCAGATGAACC | XM_021068494.1 |
|  | R:AACCTTATTTGCCACCTCCTCTGTAAC |  |
| *Mfn2* | F: CCACACCACCAACTGCTTCCTG | XM_021095371.1 |
|  | R: TCTTGACGCTCCTCTTCTCCTCTG |  |
| *OPA1* | F: ACAGAGGATGGTGCTTGTTGACTTAC | XM_021070066.1 |
|  | R: ACACAGTATGATGGCGTTGGGATTC |  |
| *Drp1* | F: TAAACCGAAGCCAGAAGGACA | XM_021069581.1 |
|  | R: AAGTGGCGATAGGAAGGGTG |  |
| *Fis1* | F: CAGACAGAGCCACAGAACAACCAG | XM_021086263.1 |
|  | R: CAAGTCCAATGAGTCCAGCCAGTC |  |
| *Nrf2* | F: CCAATTCAGCCAGCACAACACATC | XM_013984303.2 |
|  | R: GACTGAGCCTGGTTAGGAGCAATG |  |
| *Keap1* | F: GGAGGACCACACCAAGCAAGC | NM_001114671.1 |
|  | R: GGATGAAGCCAGCACCACCTTG |  |
| *NQO1* | F: GTGGAAGCCGCAGACCTTGTG | NM_001159613.1 |
|  | R: CATGGCAGCGTATGTGTAAGCAAAC |  |
| *HO-1* | F: CCAGGTCCTCAAGAAGATTGCTCAG | NM_001004027.1 |
|  | R: GGGTCATCTCCAGAGTGTTCATTCG |  |
| *IRE1α* | F: GAGCAGCCTTAACCCACACT | XM_005668695.3 |
|  | R: GTACCCGCCAGACACTCAAA |  |
| *GRP78* | F: ACCAAAATCGCCTGACACCT | XM_001927795.7 |
|  | R: TGCGCTCCTTGAGCTTTTTG |  |
| *ATF6* | F: CCGAAGAGAAGAGCCATCTG | XM_001924512.4 |
|  | R: TCCTTTGATTTGCAGGGTTC |  |
| *PERK* | F: AGACTGTGACTTGGAGGACG | XM_003124925.4 |
|  | R: GGATGCGTTATCACAGCCAG |  |
| *EIF2α* | F: TCGTCATGTTGCTGAGGTGTTGG | XM_001928339.4 |
|  | R: TGTCATCAAAGACCCAGGCAGTTC |  |
| *ATF-4* | F: TGGCGTATTAGAGGCAGCAG | NM_001123078.1 |
|  | R: TTTGTCGGTTACAGCAACGC |  |
